# Estimated Muscle Fiber Type and Blood Lactate Responses in NCAA Division III Sprint and Distance Swimmers: An Exploratory Feasibility Study

**DOI:** 10.3390/jfmk11030321

**Published:** 2026-08-19

**Authors:** Justin A. DeBlauw, Elissa Miller, Kyle D. Heise, Will K. Quinn, Stephen J. Ives

**Affiliations:** 1 Department of Health and Human Physiological Sciences, Skidmore College, Saratoga Springs, NY 12866, USA; justindeblauw@suu.edu (J.A.D.); emiller4@skidmore.edu (E.M.); kyleheise@skidmore.edu (K.D.H.); wquinn@skidmore.edu (W.K.Q.); 2 Department of Kinesiology and Outdoor Recreation, Southern Utah University, Cedar City, UT 84720, USA

**Keywords:** swimming, exercise performance, sport, anaerobic capacity, isokinetic

## Abstract

**Background and Objectives:** Swimming has sprint and distance events, with current performance often driving selection rather than physiological characteristics of the athlete (e.g., muscle fiber type, blood lactate, etc.), especially among NCAA Division III swimmers, where these characteristics are unknown. We aimed to characterize the differences in estimated muscle fiber types (% fast twitch, %FT) and blood lactate (B_lac_) response to the maximal anaerobic lactate swim test (MANLT) between collegiate distance and sprint collegiate swimmers. **Methods:** Twenty-two college-aged (19.7 ± 1.0 yr) DIII swimmers (n = 13 sprint, n = 9 distance) performed an isokinetic muscular fatigue protocol to estimate their %FT, as well as their maximal and minimum B_lac_ values and clearance rate (B_lac_ recovery) (3 and 12 min post) after the MANLT was performed on two separate days (>48 h between). **Results:** There were no significant differences between sprint swimmers and distance swimmers in maximal B_lac_ levels (11.6 ± 2.5 vs. 10.5 ± 3.4 mM, *p* = 0.38, d = 0.3), minimal B_lac_ levels (9.3 ± 2.9 vs. 8.3 ± 3.3 mM, *p* = 0.45, d = 0.3), and percent decreases from 0 to 3 min (0.9 ± 13.1 vs. 9.9 ± 9.5, *p* = 0.09, d = 0.7), 0–12 min (15.4 ± 15.6 vs. 19.7 ± %, *p* = 0.52, d = 0.2), and 3–12 min (14.7 ± 12.5 vs. 11.04 ± 11.2%, *p* = 0.48, d = 0.3) B_lac_ recovery. No significant differences were identified in estimated %FT between sprinters and distance swimmers (52 ± 6.4 vs. 45 ± 10.51, *p* = 0.056). Both extensor and flexor %FT estimations showed 69.2% and 66.7% accuracy in matching coach-identified event, with their respective estimated fiber type. A positive relation was identified between %FT knee flexor fibers and B_lac_ (r = 0.44) and between %FT knee extensor fibers and B_lac_ (r = 0.48) for all swimmers. **Conclusions:** This preliminary study suggests that athlete specialization is generally accurate to the physiology of the swimmer, namely anaerobic capacity and estimated fiber type.

## 1. Introduction

Competitive swimming is a worldwide sport, consisting of athletes of all ages and a multitude of strokes: butterfly, breaststroke, backstroke, and freestyle [1]. Swimming events are often categorized as either sprint, middle-distance or distance [1,2,3]. These categories are determined based on which energy systems are required for each specific distance [4,5]. Sprint swimming usually consists of distances that are 100 m or less, and requires short, high-intensity, explosive uses of energy that last under 60 s [6]. In contrast, distance swimming usually consists of distances that are 400 m or more, and requires long, low-intensity, controlled uses of energy that last longer than five minutes. Middle-distance swimming usually falls between the two. Swimming performance is influenced by anthropometrical, physiological, and biomechanical factors [1,2,3]. The influences of each factor have been a topic of interest in swimming research as studies have shifted from an anthropometrical and biomechanical focus to a physiological focus. While one study indicated that biomechanical factors explained most of a 100 m freestyle swim performance variability (90.3%), followed by anthropometrical (45.8%) and physiological (45.2%) parameters, a focus on physiological variables is stressed due to training adaptability and conditioning [3].

One physiological factor this study aims to explore is the distribution of muscle composition made up of slow-twitch oxidative (type I) and fast-twitch (oxidative type IIa and glycolytic type IIx) muscle fibers [5]. Slow-twitch (ST) fibers are predominantly suited for aerobic activities and repetitive submaximal contractions, as they are oxidative, fatigue-resistant, and have increased mitochondrial activity. In contrast, fast-twitch (FT) fibers are predominantly suited for anaerobic activities, as they generate high force, fatigue quicker, and rely on alactic and lactic energy systems. Understanding fiber types is important because these factors can help explain individual differences in sprint and distance performance, responses to training, and further help in designing programs best fit for the success of the athlete [7]. The previous literature suggests that sprint-oriented athletes are associated with predominantly FT fibers while distance-oriented athletes are associated with predominantly ST fibers [8]. Furthermore, swimming research has aligned with the literature, as sprint swimming ability is closely related to high relative FT fiber expression and usage of an anaerobic energy system, while distance swimming ability is closely related to high relative ST fiber expression and usage of an aerobic energy system [9]. A study done by Gerard et al. found that biopsied muscle fiber type distribution profiles matched their respective classified swimming specialty in elite sprint and long-distance swimmers [9]. To determine the makeup of specific muscle fiber typology, several different methods can be implemented.

Muscle biopsies are invasive, expensive, time-consuming, and technically challenging. Alternatively, non-invasive studies have shown that the usage of surface electromyography (sEMG) could be used for the determination of muscle fiber type [10], while there is an active debate on the accuracy and interpretation of sEMG muscle fiber typing [11]. In the absence of a muscle biopsy and sEMG, performance tests to accurately estimate muscle fiber types can be effective, rapid, and noninvasive alternatives [12,13]. For example, a protocol developed by Suter et al., consisting of a knee extension fatigue test performed on an isokinetic dynamometer, was demonstrated to be an effective way of determining relative muscle fiber distribution [13]. Using a series of noninvasive functional isokinetic testing and muscle biopsy analysis for proportion of type I vs. type II fibers (%), correlations and regression models were used to explore the relations between biopsy-derived fiber type (%type I vs. %type II) and muscle function. A fatigue test of 60 maximal contractions was used to derive an estimate of the distribution of FT types in the vastus lateralis, based on the principle that a larger drop in torque output over time would correspond with a greater proportion (%) of type II vs. type I muscle fibers [13]. The authors reported the highest correlation (r = −0.64) of %type II fibers with the relative drop in torque after 55 repetitions (T55 = torque of reps 53–55/Peak torque during test), and in the regression model, the T55 accounted for 41% of the variance in type II% fiber and the root mean square error was 8% [13]. An individual’s response to these tests can provide valuable information about their physiology and likely sprint and distance performances of swimmers, but this has yet to be tested.

Another physiological property this study aims to explore is lactate, which accumulates in the blood as blood lactate (B_lac_) and is easily measured. Lactate is a byproduct of anaerobic glycolysis and increases with intensity. B_lac_ accumulates when the body’s demand for oxygen exceeds the supply, and thus is often used as a marker of an individual’s ability to work under anaerobic conditions, such as sprinting, and the ability to clear lactate through aerobic processes [5]. Lactate plays a vital role in buffering the hydrogen ion (H^+^) buildup in the bloodstream during ATP hydrolysis [5]. B_lac_ has become the center of attention for swimming research, as lactate threshold and post-exercise lactate levels and clearance are commonly used to track swim progress and performance [4]. Several studies have shown that B_lac_ is a measure of anaerobic capacity, and it has previously been used to differentiate between sprint and distance swimmers [1,2,3,4]. Short duration, high-intensity requirements from sprint swimming have been associated with high levels of B_lac_ accumulation and accumulation rates, and lower clearance rates, while the opposite holds true for longer duration, lower-intensity requirements from distance swimming [1,2,3,4].

A common swim test performed to produce adequate amounts of lactate is the maximal anaerobic lactic test (MANLT), which, upon completion, is followed by B_lac_ measurements at different time points such as 3 min and 12 min post-test [14,15]. In one study, performed by Pelayo et al., measurements of B_lac_ concentration and performance during the MANLT during a 23-week swim season (weeks 1–10 doing aerobic training and weeks 11–23 doing anaerobic training) of six national swimmers were assessed periodically and compared [14]. The study found that % B_lac_ recovery was positively correlated with the percentage of aerobic training, while % B_lac_ recovery was negatively correlated with the percentage of anaerobic training [14]. These results suggest that % B_lac_ recovery could be an efficient marker for monitoring the impact of aerobic and anaerobic training. In an additional study conducted by Mavroudi et al., B_lac_ responses of highly trained swimmers compared to maximal effort sprints suggested that maximal B_lac_ accumulation rates were a good index of anaerobic lactic power [15]. Similarly, Lätt et al. found that change in B_lac_ accumulation was the best physiological indicator of sprint swimming performance [3]. No studies, to date, have used a combination of a fatigue test that estimates relative muscle fiber type (%fast twitch vs. slow twitch) and the MANLT that measures B_lac_ accumulation levels post-exercise to match and compare these test results to previously determined self/coach-identified sprint or distance event status in Division III college swimmer profiles.

Thus, we aimed to characterize these two physiological factors (Blac and %FT), how they are reflected in a swimmer’s sprint or distance status, and explore the association between estimated fiber type and Blac. Therefore, the purpose of this exploratory feasibility study was to characterize estimated muscle fiber type (i.e., %fast twitch) and Blac profiles between Division III collegiate self/coach-identified distance or sprint swimmers. It was hypothesized that sprint swimmers would exhibit greater relative content (%) of estimated fast-twitch muscle fibers and anaerobic capacity, consisting of a higher Blac maximum and minimum, and lower Blac percent decreases from all measurement times compared to distance swimmers. The findings of the current exploratory feasibility study can help guide future studies of larger scale by providing preliminary data and estimates of effect size and providing hypothesis-generating analyses.

## 2. Methods

### 2.1. Participants and General Procedures

Healthy college-aged male and female self/coach-identified sprint (n = 13) and distance (n = 9) swimmers on an NCAA Division III swim team were recruited to participate in this pilot study. Recruitment was carried out through Skidmore College email, word of mouth, and text for individuals who fit the experiment criteria. Participants were required to be an active member of the Skidmore College Division III Swimming team during the 2023–2024 season, present with no contraindications on a healthy history screening form, able to perform repeated maximal effort leg extensions and flexions, and complete a maximal anaerobic lactate swim test (MANLT) to fit the inclusion criteria. Exclusion criteria excluded participants with significant health conditions contraindicating physical activity and recovery, who smoked, and/or wore any implanted/internal electrical medical devices. All participants reported no physical or health limitations, as determined by a medical health history questionnaire and physical activity readiness questionnaire. Participants gave their written informed consent prior to participation. This study was performed in accordance with the Declaration of Helsinki, and all procedures were approved by the local ethics committee at Skidmore College (IRB# 2402-1131).

### 2.2. Experiment Overview

**Figure 1:**
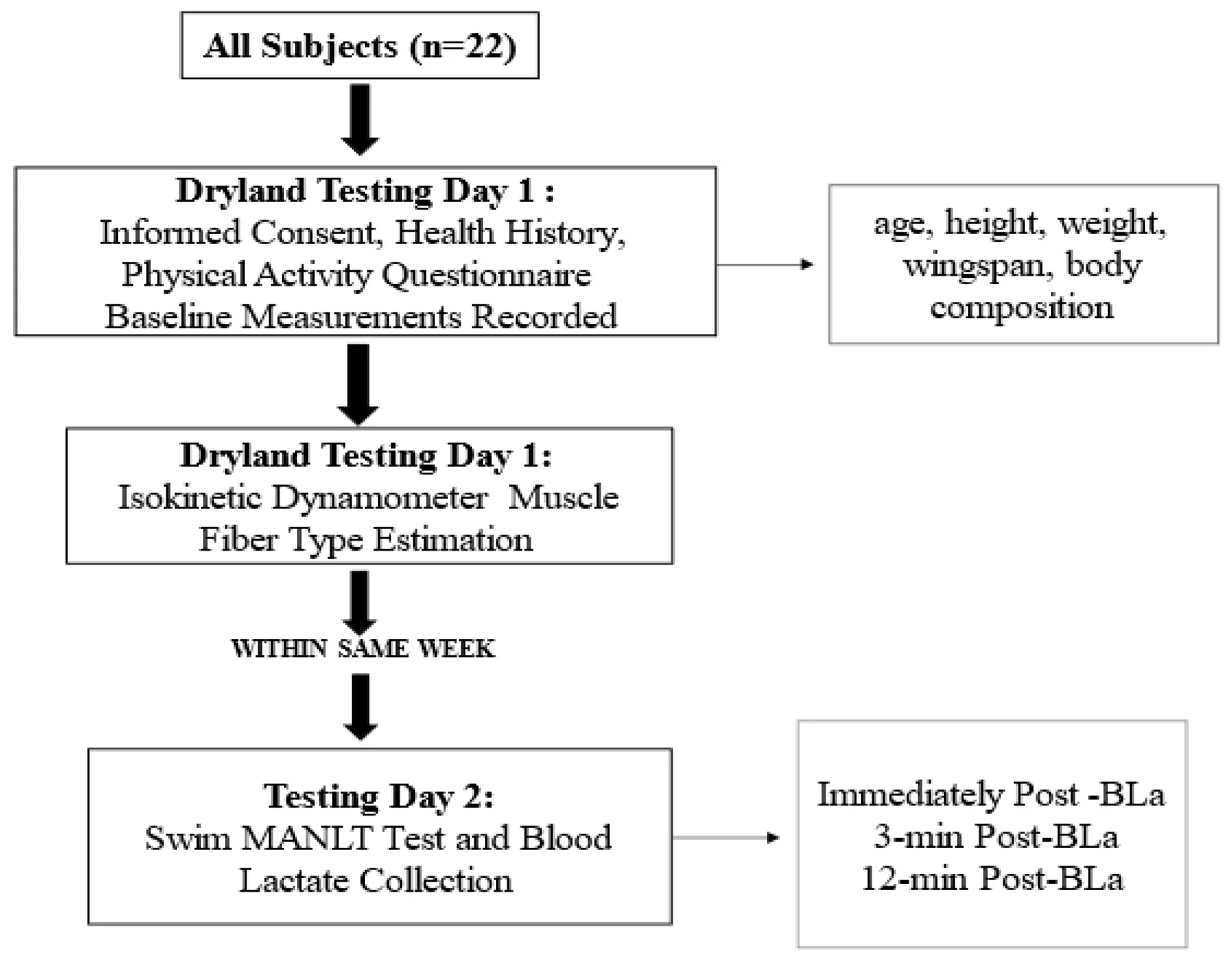
Overview of Exploratory Feasibility Study Design.

An experimental cross-sectional study analyzed if differences existed in muscle fiber types and lactate profiles between Division III collegiate distance and sprint swimmers. Prior to the beginning of data collection, all participants underwent in-person baseline testing at Skidmore College. Upon the participants’ arrival, written informed consent was obtained, and participants were screened for eligibility. Healthy college-aged male and female swimmers (n = 22, 19.7 ± 1.0 yr) performed a knee extension fatigue test on an isokinetic dynamometer and a MANLT on separate visits, respectively. Peak torque and knee extension repetitions of 53, 54, and 55 were measured and used to determine FT muscle percentage. B_lac_ (mmol/L) was measured immediately, 3 min, and 12 min post-MANLT. Figure 1 displays the experimental overview.

### 2.3. Anthropometrics, Body Composition and Self-Identification of Event Type

Baseline measurements of age (yr), height (cm), and wingspan (inch) were recorded before any testing. Age was self-reported. Height was measured to the nearest 0.01 cm using a stadiometer (Seca, Mt. Pleasant, SC, USA), and wingspan was measured to the nearest 0.01 inch using a measuring tape (Gulick II Plus, Perform Better, West Warwick, RI, USA). Total body composition variables of body mass (BM, kg), body fat percentage (BF%), muscle mass (MM, kg), and percent body water (BW%) were measured using a Tanita body composition scale (RD-545, Tanita, Arlington Heights, IL, USA). All anthropometric assessments were performed by trained members of the research team to ensure accuracy and consistency (typical in-house reliability coefficient of variation < 1%). Participants verbally informed the tester if they considered themselves a sprint or distance swimmer, and further identification was influenced by the head coach’s input.

### 2.4. Isokinetic Dynamometer Variables: Torque Values and FT Fiber Distribution

After baseline measurements were recorded, participants were asked to perform 55 single right knee extensor and flexor contractions at an angular velocity of 90°∙s^−1^ [13] while seated and secured via an HUMAC NORM Cybex isokinetic dynamometer (HUMAC Norm, Computer Sports Medicine Inc., Stoughton, MA, USA) [16,17]. Subjects were continuously encouraged to perform a maximal effort for each contraction and were provided with visual force output feedback (i.e., a screen displayed their data in real time). The means of contractions 3–5, 13–15, 23–25, 33–35, 43–45, and 53–55 were calculated and expressed as percentages of the highest torque [13]. Absolute and relative torque (T) values were measured and further used to estimate the relative fast-twitch muscle fiber percentage (%FT fibers). Briefly, this fatigue test of 55 maximal knee extension/flexion contractions was used to derive an estimate of the relative distribution of fiber types (% fast twitch vs. % slow twitch) in the vastus lateralis, based on the principle that a larger drop in torque output over time would correspond with a greater proportion (%) of type II vs. type I muscle fibers [13]. To ensure adequate performance, participants were asked to refrain from exercise 24 h prior to any and all testing.

### 2.5. Maximal Anaerobic Lactic Test (MANLT) and Blood Lactate Measurements

The swim test of the study was performed at the Skidmore College Williamson Sports Center 25-yard pool. Swimmers were instructed to perform a standard 1650-yard warm-up and cool-down swim. The MANLT [14] was performed following completion of the warm-up. Swimmers completed four sets of 50-yard freestyle all-out sprints with 10 s of rest in between each set. A 50-yard freestyle consists of two lengths back and forth. Once each 50 was completed, the swimmer was allotted 10 s of rest before they performed the next repetition. Participants were verbally encouraged to perform at maximal effort by testers and were instructed when ten seconds had passed after each 50-yard swim. Individual 50-yard swim times were recorded via stopwatch (seconds). After the completion of the MANLT, B_lac_ levels (mmol/La) were measured using a lactate analyzer (Lactate Pro, Nova Biomedical, Waltham, MA, USA) immediately following the test, 3 min after the test, and 12 min after the test. Subjects were instructed to maintain a seated passive recovery during these measurements. A pressure-activated lancet in the fingertip of the non-dominant hand was used. An amount of 0.7 µL of blood was collected for each finger stick. In our laboratory, within-day reliability of Blac measures is <2% coefficient of variation (CV). The MANLT testing procedure was adapted from Pelayo et al. to measure the maximal anaerobic contribution and rate of lactate clearance [14].

### 2.6. Triton Wear

Swimmers wore their personal Triton Wear device, a wearable accelerometer, used to track swim metrics (Triton Wear, Toronto, ON, Canada). This device was placed under the swim caps and was used by every swimmer during the swim competition season practices. Swimmers were instructed to connect their personal Triton Wear device to their cell phone via Bluetooth. The measurements analyzed from the MANLT were 50-yard swim time (s) for each 50 yd. Upon completion of the MANLT, a data file containing the swimmer’s recorded metrics was exported and downloaded for analysis.

### 2.7. Data Collection and Analysis

All statistical analyses were carried out using open-source software (Jamovi, v 2.5, Sydney, Australia) [18]. The Shapiro–Wilk and Levene’s tests of normality and assumption were conducted to see if the data were normally distributed and variances were similar between samples, respectively. Maximal and minimum Blac levels were calculated for sprint and distance swimmers as the highest (Blac Maximal) and lowest (Blac Minimum) value for each swimmer during the post-MANLT recovery period (0–12 mins). Comparisons between sprint and distance group maximal, minimum, and 0, 3, and 12 min post-test values, B_lac_ levels, B_lac_ percent recovery between 0 and 12 min, 0–3 min, and 3–12 min post-test, and %FT fiber were performed via an unpaired *t*-test. Cohen’s d was used as an estimate of effect size, and, as per tradition, effects of 0.2, 0.5, and 0.8 or greater were interpreted as small, moderate, or large effects. Pearson’s correlational analysis was conducted to explore the potential relationship between %FT fiber and B_lac_ max. The Shapiro–Wilk test of normality was conducted to see if the data were normally distributed; if a violation was found, Spearman’s rho would be used. The r value was used as an estimate of effect size, and, as per tradition, the strength of the relationship was defined as follows: r values of 0.00–0.10, 0.10–0.39, 0.40–0.69, 0.70–0.89, and 0.90–1.00 were interpreted as negligible, weak, moderate, strong, or very strong correlations, respectively. All levels of significance were set as *p* < 0.05 and used a two-tailed approach. All data are expressed as means ± standard deviation.

## 3. Results

### 3.1. Athlete Descriptive Characteristics

Descriptive characteristics of the participants at the onset of the study are displayed in Table 1. Participants’ ages ranged from 18 to 22 years, and there were nine males and 13 females who participated in this study, comprising ~50% of the team total. Participants were dichotomously grouped into either self/coach-identified sprint (n = 13) or distance swimmers (n = 9), and no significant differences in age, anthropometrics (height, wingspan), or body composition (BM, BF%, MM, and PBW%) were found between groups (all *p*-values > 0.05). All athletes successfully completed all testing sessions and thus completion was 100%, and there were no adverse events. Only 1 distance swimmer was excluded from the event-type analysis as their estimated fiber type according to knee extensor fiber type estimation was equally split 50–50, netting a ≥95% analysis of data.

### 3.2. Percentage FT Fiber Estimations

**Figure 2:**
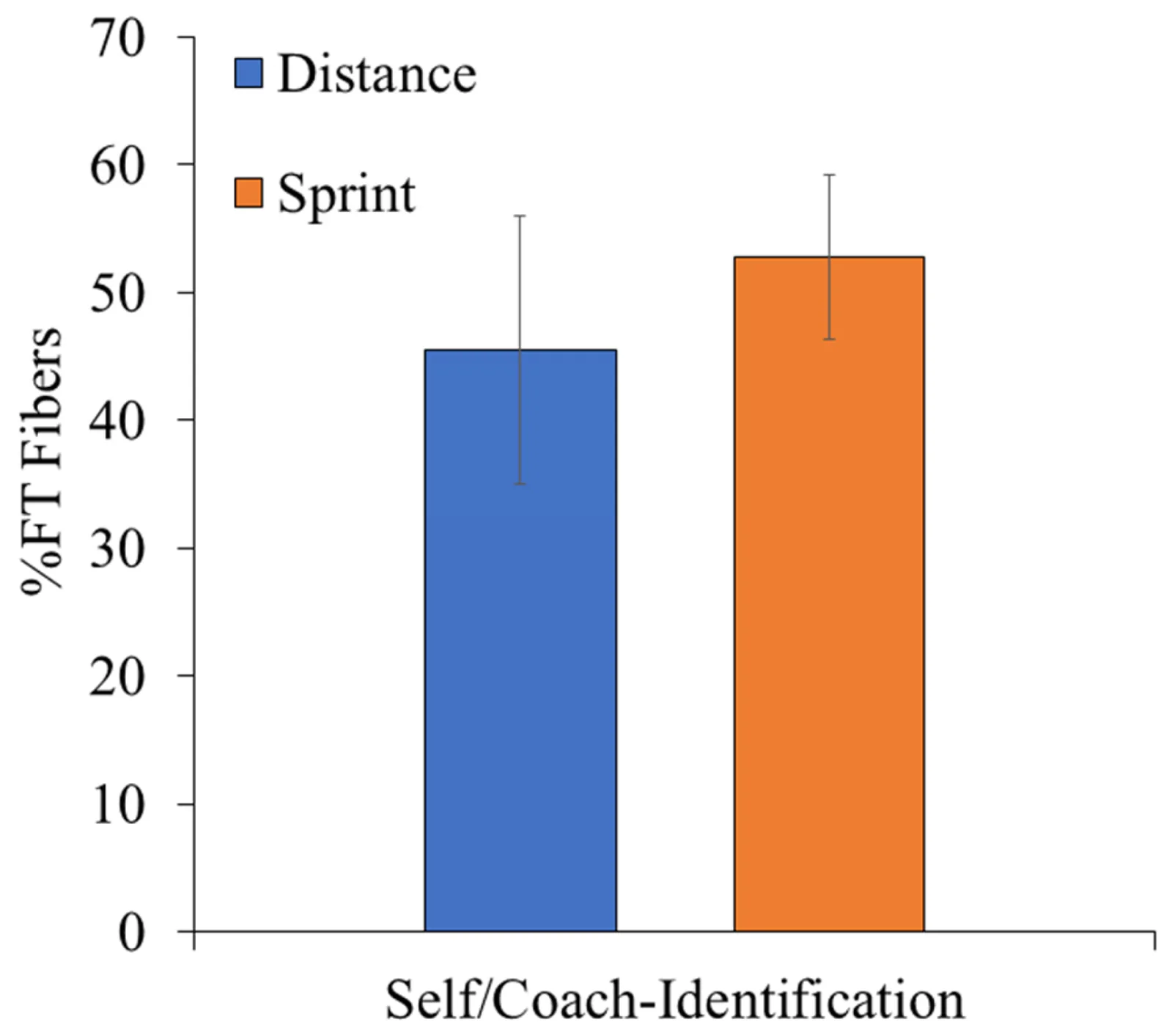
A comparison of estimated %FT Fibers between self/coach-identified distance and sprint swimmers. (distance mean 45.44 ± 10.51, sprint mean 52.77 ± 6.47, *p* = 0.056). All data are expressed as means ± standard deviation.

Table 2 displays the accuracy in self/coach-identified sprint and distance swimmers compared to their muscle fiber type percentage estimated profiles from both the knee extensor and flexor results. Nine of the thirteen self/coach-identified sprint swimmers were correctly matched with their muscle fiber type estimations for both extensor and flexor results. Six of the nine self/coach-identified distance swimmers were correctly matched with their muscle fiber type estimations for both extensor and flexor results. The accuracy in self/coach identified specialty was determined based on meeting a set criterion where performance indicating (>50% FT fibers) would classify a swimmer as a sprint swimmer, and performance indicating (>50% ST fibers) would classify a swimmer as a distance swimmer. Figure 2 displays the comparison between self/coach-identified sprint and distance groups of estimated %FT fibers, with the sprint group mean of 53 ± 7 and distance group mean of 45 ± 11% FT fibers that trended towards significance (*p* = 0.056, Figure 2).

### 3.3. Swim Performance Comparison Between Sprint and Distance Swimmers

Table 3 displays the 50 yd swim times obtained during the MANLT. There were no significant differences between the self/coach-identified sprint and distance swimmers in 50 m swim times at first, second, third, and fourth sprint intervals. On average, self/coach-identified sprint swimmers had lower 50 yd sprint times than the distance swimmers. The self/coach-identified sprint swimmers had a 15% increase in 50 yd times from the first to fourth sprint. Conversely, the self/coach-identified distance swimmers had a 6% increase in 50 yd sprint time from the first to fourth sprint. There was no significant difference (*p* = 0.99; d = 0.00) in performance decrement between groups for the total performance decrement (first interval to fourth interval).

### 3.4. Blood Lactate Comparisons Between Self/Coach-Identified Athlete-Event Matching

Table 4 displayed the B_lac_ variables obtained following the MANLT. There were no significant differences between the self/coach-identified sprint and distance group in B_lac_ maximum, B_lac_ minimum values, nor B_lac_ percent decreases during 0–12 min, 0–3 min, and 3–12 min post-test respectively (*p* = 0.385, *p* = 0.452, *p* = 0.526, *p* = 0.093, and *p* = 0.489 respectively, Table 3) and the magnitude of this effect was medium for all conditions (Cohen’s d = −0.385, −0.333, 0.280, 0.764, −0.306 respectively). There were higher means for B_lac_ max, B_lac_ min, and B_lac_ percent decreases from 3 to 12 min post-test in the sprint group compared to the distance group.

**Figure 3:**
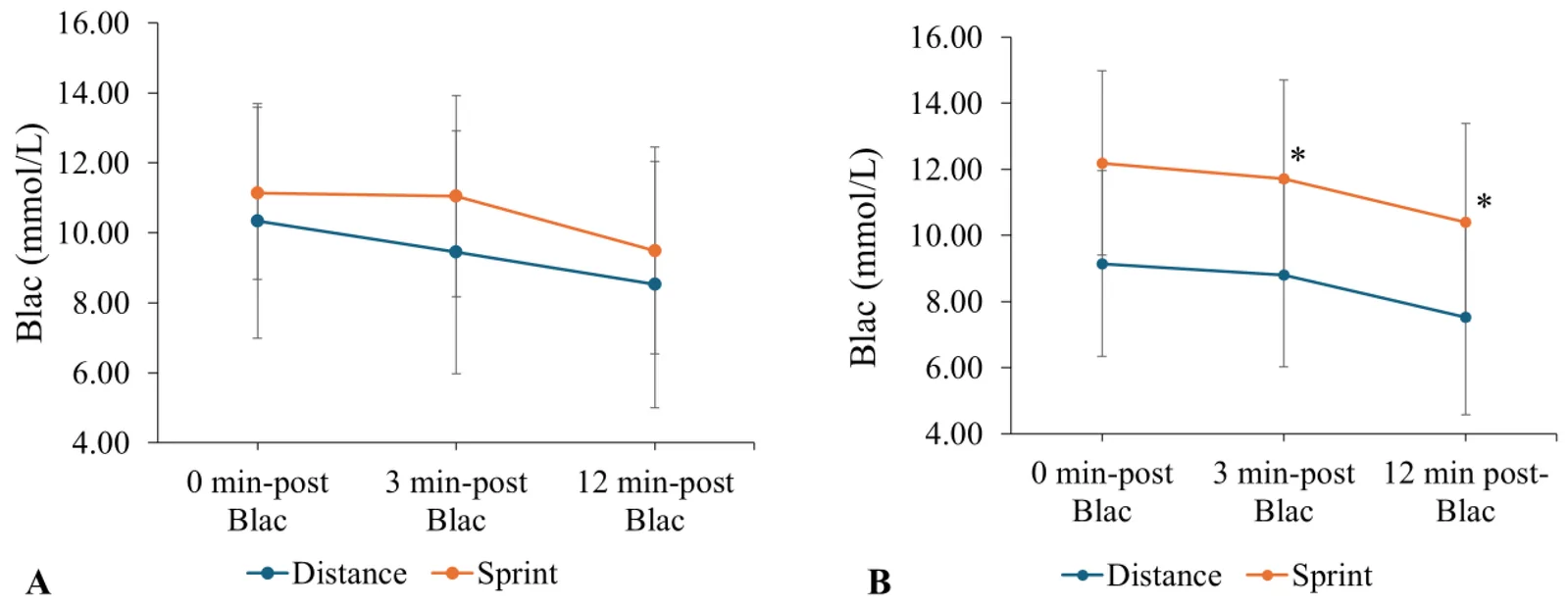
(**A**) Self/coach-identified sprint and distance swimmers’ post-MANLT B_lac_ values at 0 min (*p* = 0.53), 3 min (*p* = 0.25) and 12 min (*p* = 0.49). B_lac_ was higher in the sprint vs. distance group at all recorded minutes after MANLT. (**B**) Post Hoc Estimated Muscle Fiber Type-Based sprint and distance swimmers’ post-MANLT B_lac_ values at 0 min (*p* = 0.16), 3 min (*p* = 0.03) and 12 min (*p* = 0.05). B_lac_ was higher in the sprint vs. distance group at all recorded minutes after MANLT. All data are expressed as means ± standard deviation. * Statistically significant difference between groups (*p* ≤ 0.05).

Figure 3A shows the trend in post-test B_lac_ values at 0 min (distance mean 10.34 ± 3.35, sprint mean 11.13 ± 2.45, *p* = 0.531) 3 min (distance mean 9.44 ± 3.46, sprint mean 11.05 ± 2.88, *p* = 0.251) and 12 min (distance mean 8.52 ± 3/53, sprint mean 9.49 ± 2.95, *p* = 0.492) for self/coach-identified sprint and distance groups. Although there are greater mean values for each B_lac_ of the sprint group compared to the distance group, there were no statistical differences.

### 3.5. Muscle Group-Specific Blood Lactate Comparisons Between Sprint and Distance Swimmers

Further physiological comparisons were made in two separate post hoc analyses. Table 5 displays the B_lac_ profile differences according to extensor results of post hoc sprint (n = 13, FT % fibers > 50) and distance (n = 8, FT % fibers < 50) groups. Note that one participant had 50% FT fibers and thus was excluded from the extensor post hoc grouping. Table 5 shows no significant differences between groups in B_lac_ max values (*p* = 0.067, Cohen’s d = −0.873, Table 5). The magnitude of this effect was large. There were significant differences between groups in B_lac_ min, B_lac_ percent recovery during 0–12 min and 0–3 min post-test (*p* = 0.040, *p* = 0.044, *p* = 0.015, respectively, Cohen’s d = −0.993, 0.967, 1.207, respectively, Table 5). The magnitude of these effects was large. However, there was no difference between B_lac_ percent recovery during 3–12 min post-test (*p* = 0.700, Cohen’s d = 0.176, Table 5). The magnitude of this effect was small. Table 5 displays the B_lac_ profile differences in flexor results of post hoc sprint (n = 12, FT % fibers > 50) and distance (n = 10, FT % fibers < 50) groups. Table 6 shows significant differences between B_lac_ max and B_lac_ min values (*p* = 0.012, *p* = 0.042, respectively, Cohen’s d = −1.187, −0.931, respectively, Table 6). The magnitude of this effect was large. There were no significant differences in B_lac_ percent recovery during 0–12 min, 0–3 min, and 3–12 min post-test between the sprint and distance post hoc groups (*p* = 0.617, *p* = 0.958, and *p* = 0.590, respectively, Table 6) and the magnitude of this effect was medium, small and medium, respectively for these conditions (Cohens d = 0.2174, 0.0232, 0.2344, respectively).

Figure 3B shows the trend in post-test B_lac_ values at 0 min (sprint mean 11.53 ± 2.78, distance mean 9.71 ± 2.82, *p* = 0.16), 3 min (sprint mean 11.58 ± 3.00, distance mean 8.54 ± 2.79, *p* = 0.03) and 12 min (sprint mean 10.22 ± 2.99, distance mean 7.45 ± 2.95, *p* = 0.05) for post hoc extensor sprint and distance groups. B_lac_ was significantly higher at 3 min and 12 min in the sprint group compared to the distance group.

### 3.6. Relationships Between Fiber Types and Lactate Clearance

**Figure 4:**
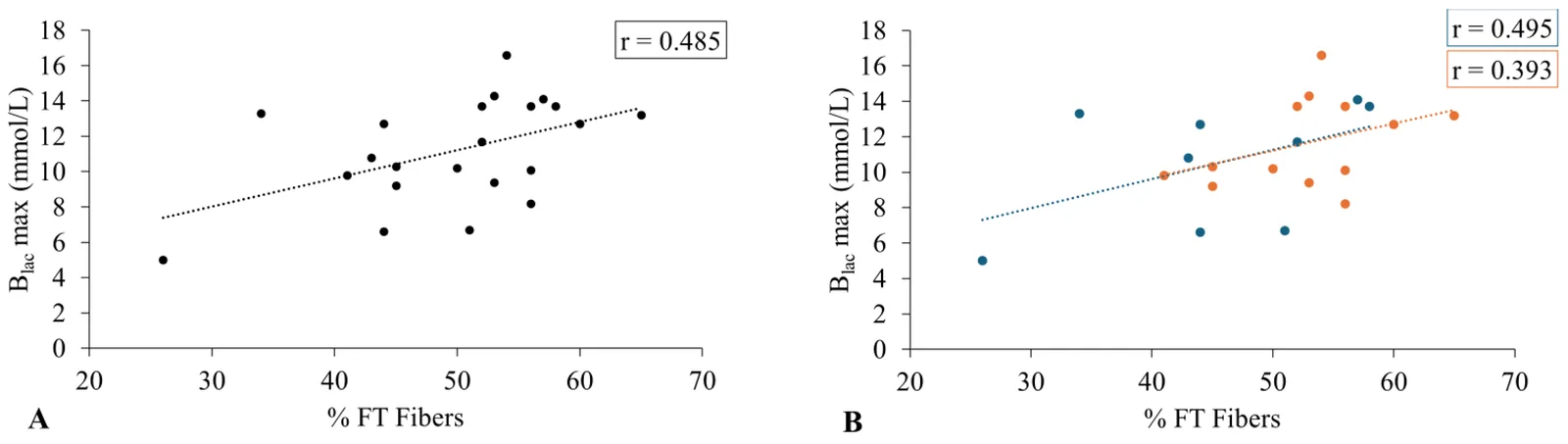
(**A**) Relationship between percentage of fast-twitch fibers (%FT fibers) based on extensor results and maximal blood lactate measurements (B_lac_ max, mmol/L) for all swimmers. (r = 0.485, n = 22). (**B**) Relationship between percentage of fast-twitch fibers (%FT fibers) based on extensor results and maximal blood lactate measurements (B_lac_ max, mmol/L) for self/coach-identified sprint (r = 0.393, n = 13, orange symbols) and distance swimmers (r = 0.495, n = 9, blue symbols). Dotted line is the line of best fit.

**Figure 5:**
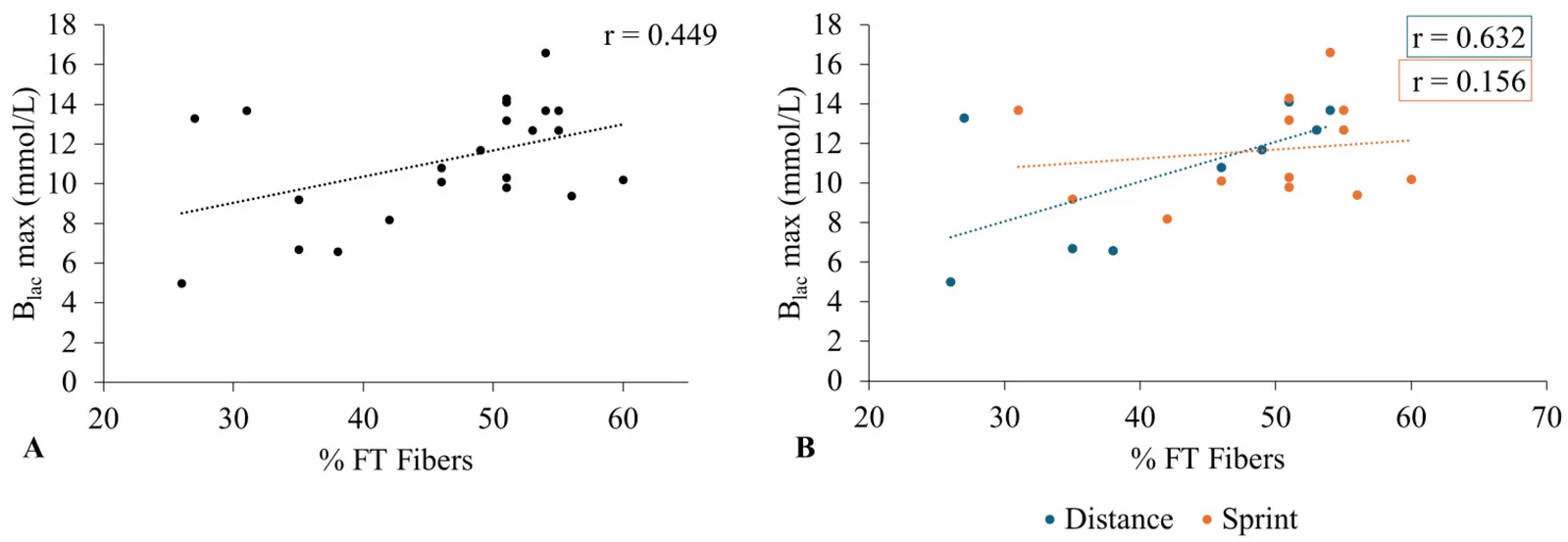
(**A**) Relationship between %FT fibers based on flexor results and B_lac_ max for all swimmers (r = 0.449, n = 22). (**B**) Relationship between %FT fibers based on flexor results and B_lac_ max for self/coach-identified sprint (r = 0.156, n = 13, orange symbols) and distance swimmers (r = 0.632, n = 9, blue symbols). Dotted line is the line of best fit.

Figure 4A displays a moderate positive relationship between %FT fibers based on extensor results and B_lac_ max for all swimmers. (r = 0.485, n = 22). Figure 4B displays a weak positive relationship between the percentage of %FT fibers based on extensor results and B_lac_ max measurements for self/coach-identified sprint (r = 0.393, n = 13) and a moderate positive relationship for distance swimmers (r = 0.495, n = 9). Figure 5A displayed a moderate positive relationship between %FT fibers based on flexor results and B_lac_ max for all swimmers (r = 0.449, n = 22). Figure 5B displayed a weak positive relationship between the percentage of %FT fibers based on flexor results and B_lac_ max measurements for self/coach-identified sprint (r = 0.156, n = 13) and a moderate positive relationship for distance swimmers (r = 0.632, n = 9).

## 4. Discussion

The purpose of this exploratory feasibility study was to characterize estimated muscle fiber types and blood lactate profiles between Division III collegiate distance and sprint swimmers. It was hypothesized that sprint swimmers would exhibit greater %FT muscle fibers and anaerobic capacity, consisting of a higher B_lac_ max and min, and lower B_lac_ percent decreases from all measurement times compared to distance swimmers. Somewhat contrary to our hypothesis, the differences between self/coach-identified sprint and distance swimmers were not significant, even if the data trended directionally towards the expected outcome, namely sprinters aligning with greater estimated %FT muscle fibers and Blac, while distance exhibited greater estimated %ST muscle fibers and lower Blac in this small single-team sample. These protocols were very well tolerated and completion was 100%, suggesting a strong likelihood of implementation. The directional patterns and moderate associations between estimated fast-twitch fiber percentage and maximal Blac warrant evaluation in a larger, prospectively designed study, and will hopefully be hypothesis-generating for such studies.

No significant differences existed between self/coach-identified groups (sprint vs. distance) in B_lac_ max (11.65 ± 2.52 mmol/L compared to 10.51 ± 3.49 mmol/L), B_lac_ min (9.36 ± 2.94 mmol/L compared to 8.33 ± 3.30 mmol/L), and B_lac_ percent recovery 0–12 min, 0–3 min, and 3–12 min-post-MANLT. Though nonsignificant, there were overall greater values for self/coach-identified sprint swimmers in B_lac_ max, B_lac_ min, and B_lac_ percent recovery 3–12 min post-MANLT. There was overall greater B_lac_ percent recovery 0–12 min and 0–3 min post-MANLT in self/coach-identified distance swimmers. Specific data pairwise comparisons did, however, yield significant differences between sprint and distance groups, as there was a trend for greater lactate clearance for distance swimmers and higher B_lac_ maximum and minimum values.

Our exploratory study suggests that overall, self/coach-identification was generally estimated in the right direction in matching athlete profiles to their expected muscle fiber type estimation (%FT). While more than half of the results from the fatigue tests indicated the expected predominant muscle fiber type percentages (e.g., greater %FT with Sprinters), there were athlete-event profiles that did not match up to their expected muscle fiber types. Such discrepancy could relate to multiple factors, such as limited precision in the estimation of muscle fiber type, event classifications and training are not purely dichotomous, and/or the onset of detraining in the post-competitive season. These potential factors are addressed in more detail below.

Distinct differences in our self/coach-identified swimmers were not as clear as the estimated fiber type classification in Figure 4B and Figure 5B. There was a weak relationship for self/coach-identified sprinters and a moderate relationship for self/coach-identified distance swimmers. However, there was also a moderate positive relationship between %FT fiber and B_lac_ maximum value from extensor data of all subjects, indicating that a trend in increased %FT fibers suggests an increase in maximal B_lac_. Further research is needed to determine the physiological parameters of swimmers during their pre-season and/or in-season training as opposed to those of swimmers in their post-season.

As previous research has shown, sprint swimmers tend to have a relatively higher proportion of FT muscle fibers (%type II), which allows for the necessary quick, explosive bursts of speed and power [9]. The opposite holds true for distance swimmers, as they tend to have a more balanced distribution of a higher proportion of slow-twitch muscle fibers (type I), which are more fatigue-resistant and efficient at utilizing oxygen. This study mostly supported this trend. However, self/coach-identification was not completely accurate in the percentage of muscle fiber types nor their expected lactate outcomes. There are several explanations for these discrepancies. For instance, the wide variety of physical activity could have influenced their performance on the isokinetic dynamometer, and thus determined their muscle fiber type composition post-season rather than what they would consider themselves to be during in-season training and competition. In addition, while swimmers were dichotomously determined to be either sprint or distance swimmers, swimmers on the swim team often considered themselves to be middle-distance swimmers, as many compete in 200-yard events.

### 4.1. Conditioning of Swimmers

All tests in this study were conducted seven to eight weeks after the swimmers’ competition season. During the 23-week competition season, swimmers regularly swam for two hours, six days per week with a combination of anaerobic and aerobic training and two hours per week of strength conditioning. During the seven to eight weeks post-competition season, swimmers partook, or did not partake, in different forms of physical activity as they were no longer regulated under a strict training schedule. The physical activity questionnaire completed at the beginning of the study indicated that the participants performed a range of moderate, vigorous, and/or strength training within the past eight weeks prior to their first test. Swimmers verbally confirmed their type of exercise as well. Of the 22 participants, only three swimmers had shown consistency in swimming training (>three days a week for ~1–2 h of aerobic training). Results from this study were expected to be influenced by two principles that could have resulted from these conditions: the detraining principle and muscle adaptations to the uncontrolled forms of exercise.

### 4.2. Detraining Principle

One explanation for the discrepancy in expected estimated muscle type distribution and lactate profiles of the self/coach-determined sprint and distance swimmers could have been due to detraining, which is the partial or complete loss of training-induced anatomical, physiological, and functional adaptations with reduced training, or even training cessation [19,20]. Participants in this study suffered from detraining. Detraining has been linked with capillarization, arterial-venous oxygen difference, and oxidative enzyme activity declines in athletes. While breaks allow for relief from the strain of training, a prolonged break may produce decrements in such variables. Specifically, energetic and kinematic adaptations may decline when training loads are decreased after the main competition seasons. It is thought that detraining may increase oxygen uptake (VO_2_) and B_lac_ concentrations for a given effort, making it more difficult to sustain the swimming speeds reached at the end of a complete season [19,20].

In a longitudinal cohort study conducted on age-group swimmers (14–15 years), researchers found that a four-week off-season detraining resulted in swimmer performance, energetic, and kinematic changes [19]. In this study, anthropometric growth and non-swimming-specific physical activities during the detraining period were controlled when comparing measurements immediately post-competition season and then measurements made after a four-week off-season period. Researchers found that lactate peak ([La^−^] peak) after detraining was higher than post-competition season measurements (*p* < 0.001; d = 0.90) [19]. Anaerobic alactic (*p* < 0.01; d = 0.99) and anaerobic lactic energy were also higher (*p* < 0.001; d = 0.92), while aerobic contribution decreased by 1.8% (*p* < 0.01; d = −1.19). Furthermore, anaerobic lactate contribution was found to increase by 1.6% (*p* < 0.01; d = 1.08). This study suggested that changes in muscle structure during cessation could be explained by decreases in mitochondrial content and function, impacting the aerobic energy pathway [19,20].

In addition, one study reported that there was a 12–18% decrease in maximal ATP production rates in isolated mitochondria following a three-week period of detraining in line with the reduction in mitochondrial respiration [21]. The decrease in mitochondrial function with training is accompanied by decreases in mitochondrial enzymes such as cytochrome c oxidase and succinate dehydrogenase at differing response rates [19,20,21]. Interestingly, mitochondrial respiration, measured via a deltoid muscle biopsy of eight male elite swimmers, decreased by 50% after only one week of inactivity and showed no further changes over the next three weeks in a study done by Costill et al. [21]. Swimmer’s physiological measurements were taken post-five months of intense training and then throughout a four-week detraining period. Throughout the detraining period, muscle glycogen decreased significantly, and post-swim B_lac_ increased significantly. However, weeks of detraining seemed to have no effect on muscle enzyme activities, and glycolytic enzymes showed an insignificant decline. In addition to these changes, Bishop et al. found that exercise-induced adaptations are quickly reversed following a reduction or cessation of physical activity, indicating muscle plasticity [20]. Overall, there is evidence to support arguments that decrements in performance and rise in post-exercise B_lac_ in weeks following cessation of exercise training are the combined result of a decline in muscle’s respiratory capacity and diminished oxygen transport system [21].

### 4.3. Unregulated Exercise

Studies have shown that training can influence muscle fiber composition and characteristics [14,22]. For example, endurance training can lead to training adaptations influencing the oxidative capacity of both fiber types, increasing their ability to effectively perform. Sprint training has also been shown to increase the size and number of fast-twitch fibers. The physical activity questionnaire indicated whether swimmers performed moderate, vigorous, and/or strength training within the past eight weeks prior to their first test. Swimmers verbally confirmed their type of exercise as well. Therefore, the wide variety of physical activity could have influenced their performance on the isokinetic dynamometer and thus determined their muscle fiber type composition post-season rather than what they would consider themselves to be during in-season training and competition.

### 4.4. Experimental Considerations

This exploratory feasibility study was limited by several factors, such as sample size, unregulated physical activity, and perceived effort on tests. The sample size used in the current study was modest (n = 22, ~50% of team total), and the disproportionate number of males compared to females could have impacted the results of the study, as there are potential sex differences in physiological profiles not accounted for. As an exploratory pilot feasibility study, this work was not designed or powered to definitively test hypotheses or establish intervention effectiveness. Rather, the findings should be interpreted as preliminary and intended to inform the design of future adequately powered studies to generate hypotheses and provide estimates of effect size. The unregulated physical activity routine of every athlete was also not controlled for. This study was conducted 7–8 weeks post the swimmers’ championship meet, where their training had been consistent and most individualized to their self/coach-identified specialty, or as a middle-distance swimmer. During the post-season, participants ranged from engaging in weight training, moderate and vigorous exercise, to no exercise at all. Their activity levels were of all different durations, intensities, and modalities. Diet, water intake, and sleep were also not regulated prior to tests, which could have impacted their test performances on the day of. Lastly, swimmers were instructed to try their hardest on each test. Some may not have been performing all tests with such intentionality. Sources of error in this study could have included inaccurate anthropometric measurements, incorrect knee positioning on the HUMAC norm, and Triton Wear malfunctions. Future studies should also document athlete experience (years or seasons of swimming) as this may play into the relation of muscle fiber type differentiation and performance.

### 4.5. Future Directions and Practical Implications for Practitioners

There has been limited research on the comparisons of muscle fiber type estimation testing and B_lac_ measurements following an all-out swimming test or race. These assessments have the potential to provide useful feedback for coaches and athletes when designing or testing the efficacy of training programs and race strategies. If confirmed by larger prospective studies, by implementing such testing during in-season training prior to major competition, swimmers will be collectively regulated and specialized, and thus their performance results would be more indicative of the success of conditioning. This is important so coaches and athletes can develop a training program tailored to maximize the genetic and physiological potential of their respective desired athlete profile. For example, if a swimmer is shown to have a naturally high proportion of FT fibers and struggles with endurance, a coach can incorporate more aerobic base training to enhance lactate clearance and improve the oxidative capacity of those fibers, but also tailor training and event selection towards sprint events for the best chances of competitive success for the individual and team. More research is needed, and in larger samples, to better understand the practical utility of the MANLT and estimation of muscle fiber type in swimmers.

## 5. Conclusions

In conclusion, in this exploratory feasibility study of a single team, we characterized the possible differences in estimated muscle fiber type (%fast twitch) between self/coach-identified sprint and distance collegiate swimmers, which was not significant, even if the data trended directionally towards the expected outcome. Future studies with larger samples could use blood lactate measurements during the MANLT and non-invasive and invasive muscle fiber typing as potential tracking methods of sprint and distance ability to adapt and respond to specific training goals. This matters because swimming comes down to being efficient and conditioned properly. Further, if blood lactate responses are indicative of muscle fiber type-specific conditioning in swimmers of sprint and distance abilities, then, by tracking these measurements, coaches may be able to improve their overall training program. The MANLT may be a good standard test to track the progress of swimmers’ physiological adaptations to pre- to post-season training, with the overall goal of benefiting an NCAA Division III swimming training program. However, further study in larger cohorts is necessary to confirm the potential practical utilization of these tests.

## Figures and Tables

**Table 1:** Anthropometrics and body composition of self/coach-identified participants.

Variables	Total (n = 22)	Sprint (n = 13)	Distance (n = 9)	*p*-Value
Age, yr	19.68 ± 1.04	19.54 ± 1.13	19.89 ± 0.93	0.45
Height, cm	172.63 ± 9.65	172.59 ± 10.65	172.68 ± 8.63	0.99
Wingspan, inch	68.88 ± 4.48	69.15 ± 4.85	68.49 ± 4.16	0.76
Body mass, kg	69.80 ± 11.28	69.59 ± 10.11	70.06 ± 13.48	0.94
Body fat percentage	22.05 ± 6.45	22.43 ± 7.60	21.40 ± 4.27	0.75
Body water percentage	58.71 ± 4.48	58.38 ± 5.23	59.27 ± 3.07	0.69
Muscle mass, kg	53.31 ± 10.21	53.94 ± 10.70	52.23 ± 10.03	0.74

All data are expressed as means ± standard deviation.

**Table 2:** Accuracy in self/coach-identified sprint and distance groups compared to knee extensor and flexor results expected muscle distribution profiles.

Subjects	Extensor	Flexors
Self/Coach-Identified Sprint (n = 13)	9/13 (69.2%)	9/13 (69.2%)
Self/Coach-Identified Distance (n = 9)	6/9 (66.7%)	6/9 (66.7%)

**Table 3:** The 50 yd swim times for sprint and distance swimmers.

Variables	Sprint (n = 13)	Distance (n = 9)	*p*-Value	Effect Size
First 50, seconds	27.78 ± 2.06	29.85 ± 2.40	0.06	0.94
Second 50, seconds	30.11 ± 2.32	31.36 ± 2.75	0.26	0.53
Third 50, seconds	31.42 ± 2.31	31.45 ± 2.60	0.90	0.05
Fourth 50, seconds	31.91 ± 2.37	31.90 ± 1.36	0.99	0.00
First decrement	−8.46 ± 3.91	−5.18 ± 6.73	0.06	0.94
Second decrement	−4.47 ± 2.03	−0.74 ± 1.84	0.26	0.53
Third decrement	−1.86 ± 3.09	−1.78 ± 5.43	0.09	0.05
Total decrement	−14.97 ± 5.24	−7.26 ± 6.57	0.99	0.00

All data are expressed as means ± standard deviation. Total decrement = performance decrement from first interval to the last.

**Table 4:** B_lac_ variables of self/coach-identified sprint and distance swimmers.

	Self/Coach Identified Sprint (n = 13)	Self/Coach Identified Distance (n = 9)	*p*-Value	Effect Size
B_lac_ Maximal	11.65 ± 2.52	10.51 ± 3.49	0.385	−0.385
B_lac_ Minimum	9.36 ± 2.94	8.33 ± 3.30	0.452	−0.333
B_lac_ decreases 0–12 min	15.46 ± 15.64	19.73 ± 14.71	0.526	0.280
B_lac_ decreases 0–3 min	0.96 ± 13.10	9.96 ± 9.50	0.093	0.764
B_lac_ decreases 3–12 min	14.72 ± 12.51	11.04 ± 11.27	0.489	−0.306

All data are expressed as means ± standard deviation.

**Table 5:** B_lac_ variables according to post hoc extensor estimated fiber type identified groups.

	Fiber Type Identified Sprint (n = 13)	Fiber Type Identified Distance (n = 8)	*p*-Value	Effect Size
B_lac_ Maximal	12.16 ± 2.80	9.71 ± 2.82	0.067	−0.873
B_lac_ Minimal	10.09 ± 2.90	7.25 ± 2.78	0.040 *	−0.993
B_lac_ decreases 0–12 min	11.62 ± 14.38	25.25 ± 13.56	0.044 *	0.967
B_lac_ decreases 0–3 min	−0.42 ± 13.24	12.94 ± 5.68	0.015 *	1.207
B_lac_ decreases 3–12 min	11.95 ± 10.12	14.11 ± 15.23	0.700	0.176

* Statistically significant difference between groups (*p* ≤ 0.05). All data are expressed as means ± standard deviation.

**Table 6:** B_lac_ variables according to post hoc flexor estimated fiber type identified groups.

	Fiber Type Identified Sprint (n = 12)	Fiber Type Identified Distance (n = 10)	*p*-Value	Effect Size
B_lac_ Maximal	12.56 ± 2.20	9.53 ± 2.93	0.012 *	−1.1865
B_lac_ Minimal	10.13 ± 2.90	7.51 ± 2.71	0.042 *	−0.9306
B_lac_ decreases 0–12 min	15.69 ± 17.28	19.02 ± 12.54	0.617	0.2174
B_lac_ decreases 0–3 min	4.51 ± 12.26	4.80 ± 13.16	0.957	0.0232
B_lac_ decreases 3–12 min	11.93 ± 13.98	14.76 ± 9.26	0.590	0.2344

* Statistically significant difference between groups (*p* ≤ 0.05). All data are expressed as means ± standard deviation.

## Data Availability

The raw data supporting the conclusions of this article will be made available by the authors on request.
